# Genistein and Glyceollin Effects on ABCC2 (MRP2) and ABCG2 (BCRP) in Caco-2 Cells

**DOI:** 10.3390/ijerph13010017

**Published:** 2015-12-22

**Authors:** Chandler Schexnayder, Robert E. Stratford

**Affiliations:** Department of Basic Pharmaceutical Sciences, College of Pharmacy, Xavier University of Louisiana, New Orleans, LA 70125, USA; cschexn2@xula.edu

**Keywords:** drug-food interaction, genistein, glyceollin, isoflavonoids, MRP2, BCRP, phytoestrogen

## Abstract

The goal of the present study was to determine the effects of glyceollins on intestinal ABCC2 (ATP Binding Cassette C2, multidrug resistance protein 2, MRP2) and ABCG2 (ATP Binding Cassette G2, breast cancer resistance protein, BCRP) function using the Caco-2 cell intestinal epithelial cell model. Glyceollins are soy-derived phytoestrogens that demonstrate anti-proliferative activity in several sources of cancer cells. 5 (and 6)-carboxy-2′,7′-dichloroflourescein (CDF) was used as a prototypical MRP2 substrate; whereas BODIPY-prazosin provided an indication of BCRP function. Comparison studies were conducted with genistein. Glyceollins were shown to inhibit MRP2-mediated CDF transport, with activity similar to the MRP2 inhibitor, MK-571. They also demonstrated concentration-dependent inhibition BCRP-mediated efflux of BODIPY-prazosin, with a potency similar to that of the recognized BCRP inhibitor, Ko143. In contrast, genistein did not appear to alter MRP2 activity and even provided a modest increase in BCRP efflux of BODIPY-prazosin. In particular, glyceollin inhibition of these two important intestinal efflux transporters suggests the potential for glyceollin to alter the absorption of other phytochemicals with which it might be co-administered as a dietary supplement, as well as alteration of the absorption of pharmaceuticals that may be administered concomitantly.

## 1. Introduction

Polyphenolic phytochemicals continue to receive attention for their cancer-preventive properties [1]. Such attention manifests in the public domain through the widespread use of dietary supplements that contain these chemicals [2] and in recommendations from public agencies to increase the consumption of fruits and vegetables because of the link between their consumption and cancer prevention [3]. In the scientific community, efforts to identify these chemicals from their multitude of sources and to evaluate their potential and underlying mechanisms to alter cell growth have been ongoing for several years [4]. A subset of phytochemicals, isoflavones, are polyphenolic compounds that resemble estrogen in their structure and biological effects [5]. Several of these are found in soy beans and red clover [6]. One compound in particular, genistein, has received much attention, including evaluation in clinical trials of various cancer types [7]. In addition to understanding the biological effects of genistein, considerable attention has been given to understanding the pharmacokinetic properties of this compound [8]. Studies have shown that genistein is highly metabolized, primarily via conjugation with sulfate and glucuronic acid, both in the intestine and liver, with consequent poor oral bioavailability of the parent molecule [9]. In addition, the transport mechanisms of genistein and its metabolites have been investigated [10,11]; these reveal a complex process of metabolism and recycling of metabolites in the gastrointestinal tract and liver via efflux transporters, including P-glycoprotein (Pgp), breast cancer resistance protein (BCRP) and multidrug resistance protein 2 (MRP2) [12].

Over the past decade, the realization that the aforementioned transporters can influence the pharmacokinetics of drugs [13] has led to efforts to systematically characterize, quantify and predict their role in drug absorption, distribution and elimination processes [14]. The participation of these efflux transporters, even as principal agents, in transporter-based drug-drug interactions is well recognized [15]. Given the role that these transporters play in the pharmacokinetics of isoflavones and many other phytochemicals, their daily consumption and the widespread use of dietary agents containing these substances, there is concern for their ability to alter drug absorption via a transporter-based drug-food interaction [16]. The presence of phytochemicals as mixtures in our diet and in food supplements has, by extension, also created interest in their interactions with one another and how such interactions may influence the safety and efficacy of these agents [17].

When soybeans are exposed to stressful conditions, such as fungal or bacterial pathogens, or UV light, they have been shown to produce glyceollins [18]. Initial studies of glyceollins’ biological effects indicated that they were estrogen receptor antagonists [19], exhibited anti-proliferative effects in estrogen-dependent breast cancer cells [20] and inhibited cell proliferation in mouse xenograft breast cancer models [21]. More recently, glyceollins demonstrated improvement in glucose homeostasis in response to a glucose challenge in a prediabetic rat model of type 2 diabetes [22] and lowered cholesterol in a hamster model of human cholesterol metabolism [23]. Overall, these pharmacologic effects have generated interest in the use of glyceollin-enriched soy as a nutri-pharmaceutical. The possibility that glyceollins would alter the transporter-based mechanism of genistein intestinal absorption, as well as that of other soy-derived isoflavones prompted our recent investigation of their potential to alter Pgp [24]. These studies demonstrated that glyceollins did not alter Pgp activity or induce Pgp expression in Caco-2 cells, thereby suggesting their inability to alter the absorption of other isoflavones, or of drugs, or of nutrients that are Pgp substrates. MRP2 and BCRP are two other important efflux transporters in the small intestine that participate in the absorption of drugs and nutrients, and are implicated in drug-drug and drug-food interactions [15]. The purpose of the study reported herein was to evaluate the potential for glyceollins to alter the function of these two transporters, again using Caco-2 as an *in vitro* model of the small intestine. To add perspective, comparison studies were conducted with genistein, since its effects on both transporters have been evaluated [25,26].

## 2. Experimental Section

### 2.1. Materials

A mixture of glyceollins I, II and III was isolated using a procedure developed at the Southern Regional Research Center (Agricultural Research Service, USDA, New Orleans, LA, USA) [19,21] and used in recent studies to evaluate glyceollin effects on glucose disposition in fat cells [22] and metabolism in rat plasma [23]. UV-VIS spectrophotometry at 285 nm allowed an estimation of the proportions of the three isomers used in all experiments: glyceollin I (68%), glyceollin II (21%) and glyceollin III (11%). Hereafter, this mixture will be referred to as “glyceollin”. Genistein was purchased from Indofine Chemical Company (Hillsborough, NJ, USA). Indomethacin was purchased from MP Biomedicals, LLC (Solon, OH, USA). Calcein acetoxymethyl ester (CAM) was purchased from AnaSpec, Inc. (Fremont, CA, USA). BODIPY-prazosin was purchased from Life Technologies, Thermo Fisher Scientific, Grand Island, NY, USA. MK-571, sodium salt, was purchased from AdipoGen, Inc. (San Diego, CA, USA). Lucifer yellow (di-lithium salt) was purchased from Thermo Fisher Scientific (Waltham, MA, USA). Except where noted in detail below, all other chemicals were purchased from Sigma-Aldrich (St. Louis, MO, USA).

### 2.2. Methods

#### 2.2.1. Cell Culture

Caco-2 cells were obtained from ATCC (Manassas, VA, USA) at Passage 27. Cells were maintained in stock cultures in Dulbecco’s Modified Eagle’s Medium (Life Technologies, Thermo Fisher Scientific, Waltham, MA, USA) supplemented with 10% fetal bovine serum (HyClone, Thermo Fisher Scientific, Waltham, MA, USA), 1% MEM non-essential amino acids (100×, Mediatech, Inc. Manassas, VA, USA), 1 mM sodium pyruvate (Mediatech), 2 mM l-glutamine (Mediatech) and 1% antibiotic/antimycotic solution (Mediatech) at 37 °C in a humidified incubator with 5% CO_2_. Stock cultures were fed 3-times per week and passed weekly. For 24-well plate uptake assays (5 (and 6)-carboxy-2′,7′-dichloroflourescein (CDF), calcein and BODIPY-prazosin), cells were seeded at 2 × 10^5^ cells/mL. Cells were seeded onto collagen coated 0.4 µm-PTFE Transwell-COL^®^ permeable supports (12 mm/12-well/1.12 cm^2^ for CDF transport studies) at a seeding density of 1.2 × 10^5^ cells/mL and cultured for 2.5 to 3.5 weeks before use. For all assays, cells were used between Passages 35 and 55.

#### 2.2.2. Effects of Glyceollin and Genistein on CDF Transport

The apical-to-basolateral (AB) and basolateral-to-apical (BA) directional transport of 5 µM CDF, an MRP2 (an efflux transporter localized to the apical membrane) and MRP3 (an efflux transporter localized to the basolateral membrane) substrate [27], in the absence *vs.* presence of 100 µM MK-571, the MRP2 inhibitor positive control [28], glyceollin or genistein, were evaluated. A 5 mM stock solution of CDFDA, the non-fluorescent di-acetate (DA) ester prodrug of CDF, was prepared in DMSO and diluted to 50 µM in pH 7.4 transport buffer (25 mM HEPES, 5 mM glucose, 1 mM NaH_2_PO_4_, 145 mM NaCl, 1 mM CaCl_2_, 0.5 mM MgCl_2_). Prior to CDFDA incubation, cells on Transwell-COL^®^ supports were washed twice with 37 °C transport buffer, incubated for 30 min at 37 °C with an inhibitor or, in the case of controls, an equivalent concentration of co-solvent (0.1% DMSO/0.1% ethanol), on both sides of the filter, and their transepithelial electrical resistance (TEER) measured (EVOM^2^ meter, World Precision Instruments, Sarasota, FL, USA). Filters with TEER values <200 Ohm-cm^2^ were not used in an incubation. In separate experiments, these concentrations of MK-571 and genistein had no effect on the permeability of Lucifer yellow, a marker of monolayer integrity, relative to controls. However, for glyceollin, Lucifer yellow permeability was increased on average 4.5-fold, from 1.3 ± 0.61 × 10^−6^ cm/s to 5.9 ± 0.37 × 10^−6^ cm/s (mean ± standard deviation). Following the addition of CDFDA to the donor compartment, filters were placed on an orbital shaker (150 rpm) set at 37 °C. Receiver compartment samples (0.4 mL) were taken every 30 min with volume replacement for 120 min for analysis of cumulative CDF mass transport in either the AB or BA transport directions. Initial and final donor samples were taken for the determination of mass balance, which was always >95%. In addition, at the conclusion of the 120-min incubation, cells on filters were washed three times with 0.5 mL of ice-cold HEPES transport buffer; subsequently, 0.2 mL of methanol were added, and the filters were incubated at 37 °C for 5 min and then sampled for determination of the CDF concentration. The CDF concentration was based on fluorescence measured in a microplate fluorescence reader (Biotek Synergy 4, Winooski, VT, USA) set at 485 nm/530 nm for excitation and emission wavelengths, respectively. Standards for CDF ranged from 7.8 to 1000 nM with *r*^2^ > 0.999. Chemical hydrolysis of CDFDA to CDF at 37 °C in the pH 7.4 transport buffer was determined to be <1% in 120 min (the details of the methodology are found below in Section 2.2.4).

Calculation of the CDF permeability coefficient was based on the following equation, where P = permeability in cm/s, *J* is the flux (the slope of the cumulative amount of CDF transported *vs.* time, expressed as nmole/s), SA is the Transwell^®^ support surface area (1.12 cm^2^) and C is the average of the initial and final donor concentrations (nmole/mL) of CDF in the form of CDFDA [13]. To clarify the terms, when CDF transport is in the AB direction, the “donor” compartment is the apical side of the cells and the “receiver” compartment is the basolateral side of the cells. These “donor” and “receiver” designations are reversed for transport in the BA direction.

P=J(SA·C)

Efflux clearance of CDF was calculated using the following equation, where *J* is as defined above, *A_cell_* represents the amount (nmoles) of CDF in the cells at the conclusion of the 120-min incubation and *V_monolayer_* represents the volume (mL) of cells on a filter. The monolayer volume was calculated as the product of the height of a typical Caco-2 cell (25 µm) [29] and the Transwell^®^ insert surface area (1.12 cm^2^).

$$Cl_{efflux}=\frac{J}{A_{cell}}\;\times \;V_{monolayer}\;\times \;\frac{60\;s}{\min}$$

#### 2.2.3. Effects of Glyceollin and Genistein on CDF and Calcein Uptake

Net uptake of CDF following exposure to 5 µM CDFDA or uptake of the fluorescent probe, calcein, which is also an MRP2 substrate [30], following exposure to 0.5 µM of its acetoxy-methyl ester (CAM), into cells grown on 24-well plates was also used to determine if genistein or glyceollin altered MRP2 activity. Following a 30-min pre-incubation in the presence of a test compound, Caco-2 cells between 75% and 95% confluency were exposed to CDFDA or CAM for 30 min, respectively, at 37 °C. The concentration range of the three compounds evaluated, glyceollin, genistein and MK-571 (positive control), was 1 to 100 µM. At the conclusion of an incubation, cells were washed 3 times in ice-cold transport buffer and then lysed in 0.1% triton X-100 (15 min at 37 °C). Subsequently, CDF or calcein fluorescence was measured in a microplate fluorescence reader at 485 nm/530 nm (excitation/emission wavelengths). Measured fluorescence was normalized to protein content based on the BCA protein assay (ThermoScientific). Bovine serum albumin was used as a reference protein, and standards were prepared over the concentration range 50 to 1600 µg/mL (*r*^2^ > 0.99 for the absorbance *vs.* albumin standard concentration relationship). In the case of CAM incubations, protein normalized net fluorescence as a function of compound concentration was fit to the following equation to estimate EC_50_ (the concentration (C) of calcein that achieves 50% of the maximum effect) and E_max_ (the maximum estimated effect), and E_0_ is the effect observed in the absence of compound, which was set to 100%: E = E_0_ + (E_max_ × C/(EC_50_ + C)).

#### 2.2.4. Effects Glyceollin and Genistein on CDFDA Hydrolysis by Caco-2 Cells

The effects of 100 µM MK-571, glyceollin or genistein on CDFDA hydrolysis to CDF were evaluated following harvest of Caco-2 cells grown for one week in two T-75 stock culture flasks. Briefly, cells at approximately 90% confluency were washed in ice-cold HEPES transport buffer twice before adding 2 mL of ice-cold 100 mM sodium phosphate buffer, pH 7.4. Pooled cell scrapings were homogenized for 60 s by hand on ice using a glass-Teflon homogenizer; subsequently, they were centrifuged at 4000× g for 5 min under refrigerated conditions. The supernatant was collected and used immediately for hydrolysis studies. Protein content was measured using the BCA protein assay as previously described for CDF and calcein uptake studies. Incubations were conducted in triplicate for each compound and control (co-solvents only) for 0, 15, 30, 60, 120 and 240 min. Non-enzymatic hydrolysis was also evaluated under control conditions. Incubations were conducted in a final volume of 200 µL (50 µL of cell preparation or buffer blank). The final concentration of CDFDA was 5 µM following the addition of 2 µL of 500 µM CDFDA stock prepared in DMSO. Incubations were terminated via the addition of 200 µL of acetonitrile and centrifugation at 4 °C at 4000 × g for 2 min under refrigerated conditions. Each 100-µL aliquot of the resultant supernatant was transferred to a 96-well plate and fluorescence measured at 485/530 nm. Net fluorescence was converted to nM CDF based on the CDF standards prepared in a 1:1 incubation matrix:acetonitrile over the concentration range 3.91 to 500 nM.

#### 2.2.5. Effects on Glyceollin and Genistein on BODIPY-Prazosin Uptake

Uptake of BODIPY-prazosin, a BCRP substrate [31] into cells grown on 24-well plates, was used to determine genistein and glyceollin effects on BCRP. Following three washes with 37 °C transport buffer and a subsequent 30-min pre-incubation in the presence of a test compound, Caco-2 cells between 80% and 95% confluency were exposed to 5 µM BODIPY-prazosin for 30 min at 37 °C. The concentration range of the three compounds evaluated, Ko143, the positive control for BCRP inhibition [32], glyceollin and genistein, was 1 to 100 µM. At the conclusion of the incubation, cells were washed 3 times in ice-cold transport buffer and then lysed in 0.1% triton X-100 (15 min at 37 °C). Subsequently, fluorescence was measured in a microplate fluorescence reader at 485 nm/530 nm (excitation/emission wavelengths). Measured fluorescence was normalized to protein content based on the BCA protein assay as described above for CDF and calcein uptake studies. For Ko143 and glyceollin, protein normalized net fluorescence as a function of compound concentration was fit to the same equation described for the analysis of the inhibition of MRP2-mediated calcein efflux. For genistein, the reduction in uptake was modeled using the following equation, where E_0_ is the effect observed in the absence of genistein, E_max_ is the estimated maximum reduction in uptake and EC_50_ is the concentration of genistein (C) that yields uptake that is 50% of E_0_: E = E_0_ – (E_max_ × C/(EC_50_ + C)).

#### 2.2.6. Data Analysis

EC_50_ estimates for the alteration of calcein or BODIPY-prazosin uptake were calculated using Phoenix WinNonlin 6.3 (Pharsight Corporation, Mountain View, CA, USA). Statistical analyses were conducted using GraphPad Prism (Prism 6 for Windows, GraphPad Software, Inc., La Jolla, CA, USA). Results shown in figures are expressed as the mean ± the standard error of the mean (SEM). An unpaired two-tailed Student’s *t*-test was used to compare two means. One-way analysis of variance, followed by, if appropriate, multiple comparison testing (Tukey’s), was used to determine if there were statistically-significant differences (defined as *p* < 0.05 unless otherwise noted) between multiple groups in an experiment.

## 3. Results and Discussion

ATP Binding Cassette (ABC) transporters are known to mediate the resistance of cancer cells to several anti-cancer drugs. These transporters are also present in normal cells throughout the body, where they function to limit cellular exposure to xenobiotics. In small intestinal epithelial cells, Pgp, BCRP and MRP2 are important in this regard [33]. Drugs that are particularly sensitive to their effects are those that have limited water solubility, so-called Class 2 drugs according to the Biopharmaceutics Drug Disposition Classification System (BDDCS) [34]. The intestinal absorption of such drugs can also be improved if they are administered in the presence of an inhibitor of any one of these three transporters. For Pgp, there are several examples of this transporter-based drug-drug interaction [35,36,37]. Phytochemicals can also alter Pgp-mediated transport [38]. In a previous study [24], we determined that glyceollin did not alter the transport of Rhodamine 123 (R123), a fluorescent dye that is a Pgp substrate [39]. In that same study, genistein was shown to actually enhance Pgp-mediated transport of R123, a finding consistent with another study [40]. The objective of the studies conducted in the present report was to expand the evaluation of glyceollin’s potential to alter intestinal transporter activity by determining its effects on MRP2 and BCRP. Companion studies were conducted with genistein.

### 3.1. Effects on MRP2 Activity

CDF is an established MRP2 and MRP3 substrate [27]. However, it is also an OATP (Organic Anion-Transporting Polypeptide) substrate and may be a specific substrate of OATP2B1 that is expressed on intestinal brush border membranes, including Caco-2 cells [41]. In order to isolate it as an MRP2/3 probe substrate, its di-acetate ester prodrug, CDFDA, which is readily permeable into cells via passive diffusion, was used [27]. Subsequent hydrolysis by cellular esterases converts the non-fluorescent CDFDA to the fluorescent MRP2/3 probe substrate.

**Figure 1:**
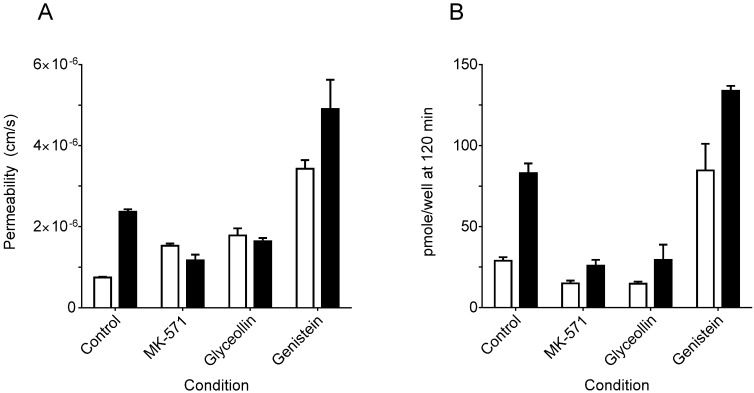
5 (and 6)-carboxy-2′,7′-dichloroflourescein (CDF) permeability in Caco-2 cells grown on Transwell^®^ filters. (**A**) CDF permeability; (**B**) amount of CDF in cells at 120 min. The condition refers to the absence of a putative inhibitor (control) *vs.* the presence of 100 µM of either MK-571, glyceollin or genistein. Open bars represent transport in the apical-to-basolateral (AB) direction and closed bars in the basolateral-to-apical (BA) direction. N = 16 experiments for controls and 3 experiments for each compound. An experiment consisted of three filters in each transport direction. For all three compounds, both permeability and cell amount measures were significantly different from the corresponding control/transport direction according to one-way ANOVA followed by Tukey’s *t*-test for multiple comparisons (*p* < 0.05).

In CDFDA/CDF transport experiments, CDF permeability was on average 3.7 ± 0.36-fold greater in the BA direction *vs.* the AB direction, reflecting a net secretory transport of CDF. The permeability ratio was significantly reduced to 0.8 ± 0.16 when both sides of the filters were exposed to 100 µM MK-571, an MRP2 inhibitor [28]. The loss in net secretory permeability was as a result of an increase in AB permeability concomitant with a decrease in BA permeability (Figure 1A). This pattern is consistent with MK-571 inhibition of MRP2-mediated CDF transport. In the case of 100 µM glyceollin, the ratio was reduced to a similar extent, 0.9 ± 0.10. As with MK-571, this reduction in the BA/AB ratio for glyceollin was due to a combination of enhanced transport in the AB direction and reduced BA directional transport, suggesting that glyceollin also inhibited MRP2. The BA/AB ratio was also significantly reduced in the presence of 100 µM genistein (2.3 ± 0.37), but not to the same extent. In the case of genistein, both AB and BA permeability were increased relative to the control (Figure 1A). A consistent pattern of cell-associated CDF, determined at 120 min, was observed (Figure 1B), in that pmole/well of CDF was consistently greater when transport was conducted in the BA direction, regardless of the control or a given compound treatment. However, relative to the control, cell-associated CDF was reduced for MK-571 and glyceollin in either direction; whereas, similar to permeability findings, pmole/well was increased in both directions relative to the control in the case of genistein treatment.

The existence of the approximate four-fold asymmetry in BA relative to AB permeability suggests that MRP2-mediated CDF efflux on the apical side was more influential than MRP3-mediated efflux on the basolateral side in determining net CDF transport in control experiments. However, the use of CDFDA in these transport experiments complicates the interpretation of the results, because delivery of CDFDA to the cells and subsequent hydrolysis to CDF, which is the actual MRP2/3 substrate, need also to be considered. Therefore, a more direct measure of the contribution of both MRP2 and MRP3 to CDF transport is to adjust the measured transport rate (flux) by the intracellular CDF concentration. Since transport was linear between 30 and 120 min (data no shown), transport kinetics were at steady state; therefore, CDF cellular concentrations measured at 120 min were assumed to be reflective of concentrations over the course of the sampled time points, beginning at 30 min. Dividing measured flux by intracellular CDF concentration yields the clearance of CDF transport. The appropriateness of this approach is supported by the approximate three-fold higher CDF concentrations following basolateral CDFDA exposure (83 ± 6.0 pmoles/filter) *vs.* apical CDFDA exposure (29 ± 2.2 pmoles/filter) observed in control incubations (Figure 1B). This difference in intracellular CDF concentration is attributed to the three-fold higher amount of CDFDA used in the BA direction due to a 1.5-mL volume on the basolateral side *vs.* a 0.5-mL volume on the apical side (75 nmole *vs.* 25 nmole of CDFDA, respectively, given the same concentration of 5 µM used in all experiments).

**Figure 2:**
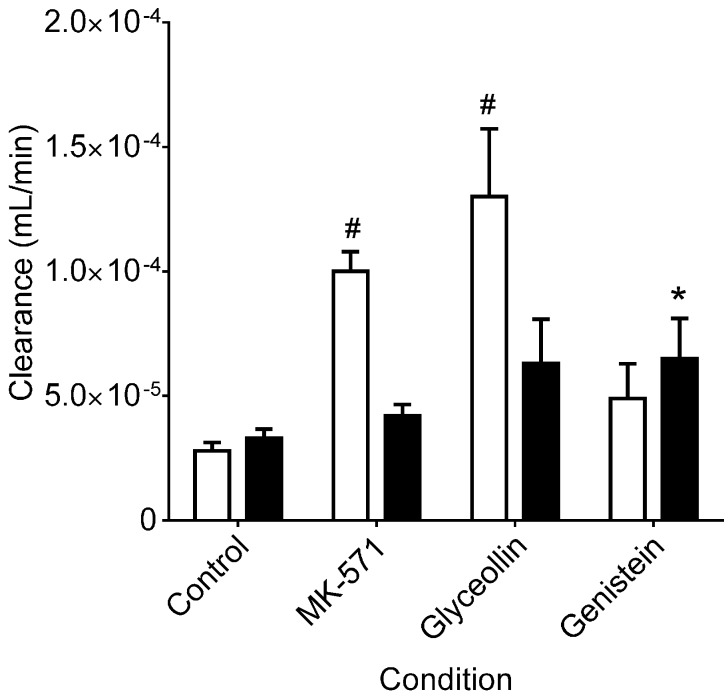
CDF clearance in Caco-2 cells. Clearance values are based on the same experiments summarized in Figure 1, with normalization of permeability by cell concentrations measured at the conclusion of each 120-min incubation. Open bars represent transport in the AB direction and closed bars in the BA direction. * and ^#^ indicate significantly different from the corresponding control/transport direction, *p* < 0.05 and *p* < 0.01, respectively, according to one-way ANOVA followed by Tukey’s *t*-test for multiple comparisons. For MK-571 and glyceollin, AB directional clearance was greater than BA directional clearance (*p* < 0.05); there was no such difference for controls and genistein.

**Figure 3:**
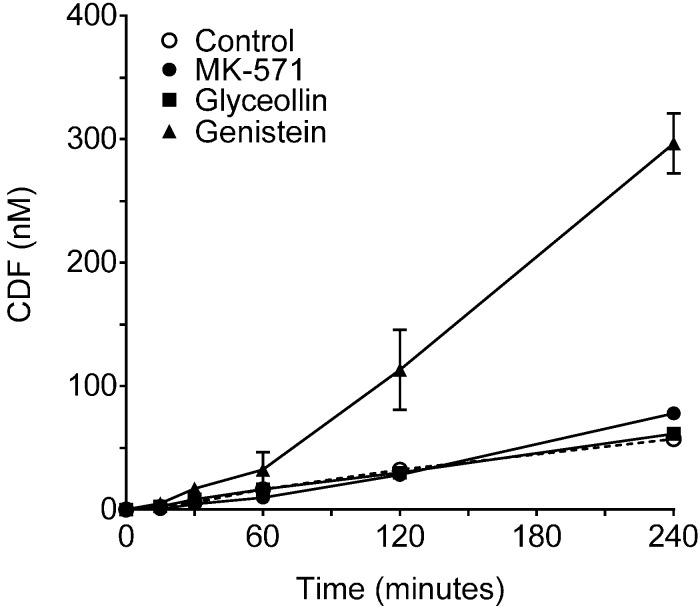
Hydrolysis of CDFDA by Caco-2 Cells. CDF concentration as a function of time following incubation with 5 µM CDFDA for up to 4 h in the absence (control, dashed line) or presence of 100 µM of MK-571, glyceollin or genistein. N = 3 for each condition. Specific activities based on initial rate conditions were 6.0, 6.2, 8.5 and 32.7 nM/min/mg protein for control, glyceollin, MK-571 and genistein, respectively.

As shown in Figure 2, in control incubations, CDF clearance into the receiver compartment was similar for the two transport directions, with a ratio of BA/AB clearance of 1.2 ± 0.07, indicating similar clearance in both directions (MRP2 mediated across the apical membrane in the BA direction and MRP3 mediated across the basolateral membrane in the AB direction), and is consistent with CDF being a substrate for both efflux transporters in Caco-2 cells. Therefore, this clearance analysis of CDF transport in Caco-2 cells yields a different conclusion than the permeability analysis and is attributed to the confounding influence of the obligatory CDFDA uptake and hydrolysis prior to CDF efflux, which are not taken into account in the permeability analysis. BA/AB clearance ratios for MK-571 and glyceollin were 0.4 ± 0.05 and 0.5 ± 0.05. These reductions were due to increased clearance across the basolateral membrane (AB directional transport, MRP3 mediated) relative to the control, with there being no significant change in clearance across the apical membrane (BA directional transport, MRP2 mediated) relative to the control (Figure 2). The increase in MRP3-mediated transport is similar to what has been observed in hepatocytes: upon inhibition of MRP2, MRP3-mediated transport of CDF is remarkably increased [27]. Importantly, CDFDA hydrolysis studies (Figure 3) indicated that neither MK-571 nor glyceollin altered the rate of hydrolysis relative to the control, thus ruling out alteration of this step in the supply of CDF following CDFDA exposure. Indeed, evaluation of CDFDA hydrolysis by Caco-2 (Figure 3) revealed that, in the presence of 100 µM MK-571 or glyceollin, the CDFDA hydrolysis rate was similar to the control (8.5 and 6.2 nM/min/mg protein, respectively, for MK-571 and glyceollin) *vs.* 6.0 nM/min/mg protein in control incubations.

**Figure 4:**
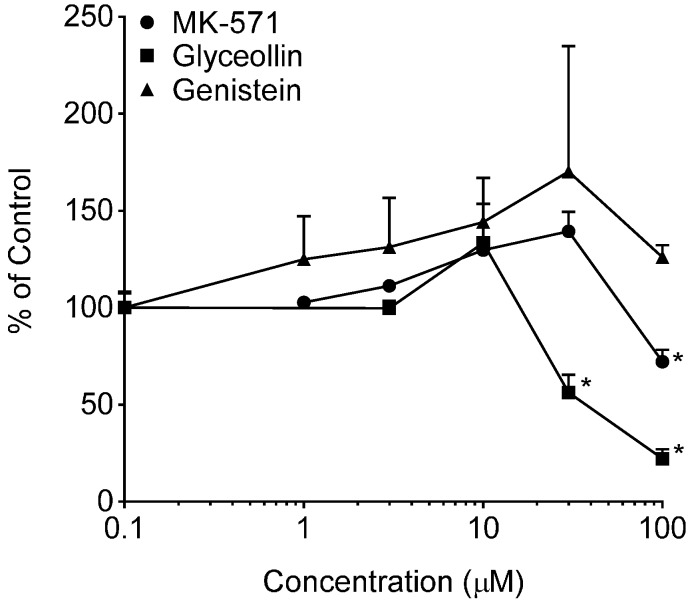
CDF uptake into Caco-2 cells. Net CDF fluorescence normalized to cell protein was measured following a 30-min incubation with 5 µM CDFDA in the presence of varying concentrations of MK-571, glyceollin or genistein ranging from 0 µM (control, arbitrarily represented as 0.1 µM on the log-scale) to 100 µM. Results represent the mean + SEM of two (glyceollin and genistein) or four (MK-571) separate experiments, with N = 4 for each concentration per experiment. * indicates significantly different from the respective compound control (*p* < 0.05) from a one-way ANOVA followed by Tukey’s *t*-test.

**Figure 5:**
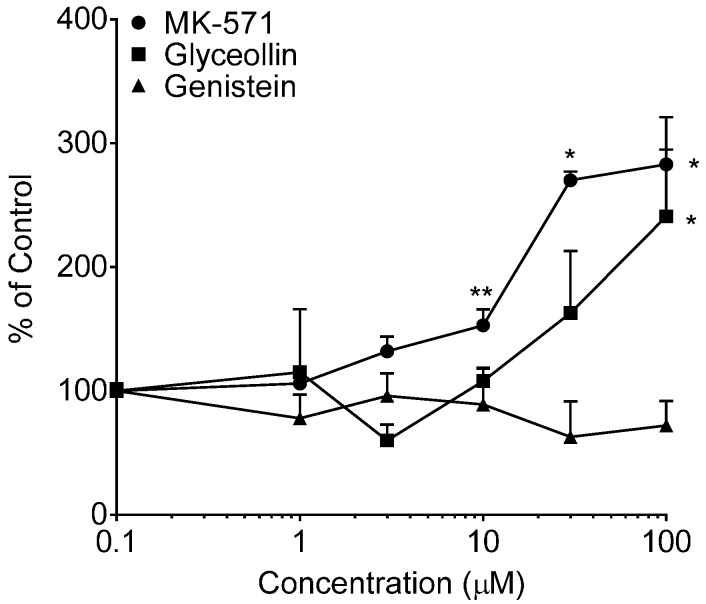
Calcein uptake in Caco-2 cells. Net fluorescence normalized to cell protein was measured following a 30-min incubation with 0.5 µM CAM in the presence of varying concentrations of MK-571, glyceollin or genistein from 0 µM (control, arbitrarily represented as 0.1 µM on the log-scale) to 100 µM. Results are expressed as the mean + SEM from two separate experiments (MK-571) or three separate experiments (glyceollin and genistein), with *N* = 4 for each concentration per experiment. * indicates significantly different from the respective compound control (*p* < 0.05) from a one-way ANOVA followed by Tukey’s *t*-test; ** indicates significantly different from the control, 30 and 100 µM conditions.

Further support for glyceollin inhibition of MRP2, and not MRP3, is based on the results of CDF retention following 30 min of exposure to CDFDA (Figure 4) and increased calcein retention following 30 min of exposure to calcein-AM (Figure 5). The absence of an increase in CDF uptake into cells in the presence of either MK-571 (a selective MRP1 and MRP2 inhibitor) or glyceollin over the range 1 to 100 µM is attributed to the retention of functional MRP3-mediated transport of CDF; in fact, the reduction of uptake observed at 100 µM may be due to enhanced MRP-3 activity that, as previously mentioned, can occur in cells upon inhibition of MRP-2 [27]. Calcein results for MK-571 demonstrated a concentration-dependent increase in uptake over the range 1 to 100 µM and were attributed to enhanced retention as a result of MRP2 inhibition. The estimated EC_50_ (concentration at half-maximal effect) was 20 µM (50% CV). Glyceollin demonstrated a trend towards a concentration-dependent increase in calcein retention over this same range; however, only the top concentration was significantly different from the control and the 3 and 10 µM conditions. It was not possible to evaluate glyceollin concentrations above 100 µM due to insolubility. There are reports that calcein is also an MRP1 substrate [42]; thus, it is possible that glyceollin could also be inhibiting MRP1-mediated calcein transport.

As indicated, the effect of genistein on CDF permeability demonstrated an increase in transport in both directions. Interestingly, CDF cellular concentrations were significantly higher in the presence of genistein (Figure 1B). These higher concentrations are attributed to a 5.5-fold increase in the CDFDA hydrolysis rate in the presence of 100 µM genistein (Figure 4; hydrolytic rate being 32.7 *vs.* 6.0 nM/min/mg protein, respectively, for genistein *vs.* control). Accounting for these higher cellular concentrations, clearance of CDF in the presence of genistein was similar in both transport directions, with a resultant BA/AB clearance ratio of 1.4 ± 0.08, which was not different from the control. However, in terms of the individual directional clearances, while genistein was not different from controls in the AB direction, clearance was slightly higher in the BA direction (Figure 2). The latter finding may indicate a slight enhancement of MRP2 activity by genistein. Similar enhancement of Pgp transport by genistein [40], and of BCRP function (this report) have also been observed. Additional evidence that genistein effects on MRP2 are slight to even non-existent are based on a lack of an effect on CDF uptake over the 1 to 100 µM concentration range (Figure 3) and a similar lack of an effect on calcein uptake over this concentration range (Figure 5). *In vivo*, genistein has been reported to inhibit MRP2-mediated hepatobiliary secretion of anionic substrates, and it has been suggested that its phase II metabolites were responsible for this [25]. Perhaps the lack of an inhibitory effect observed in Caco-2 is a result of the lower metabolic activity (specifically the genistein metabolite formation rate) in this *in vitro* model relative to *in vivo* approaches.

### 3.2. Effects on BCRP Activity

**Figure 6:**
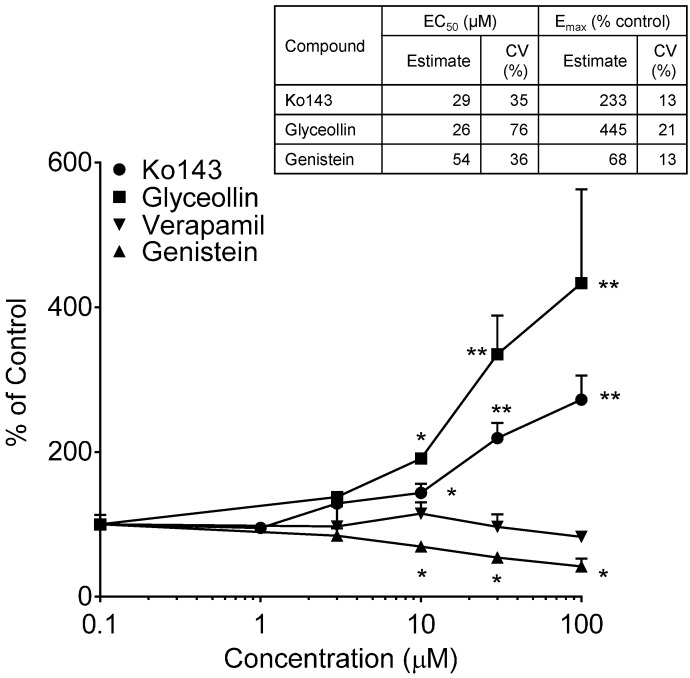
BODIPY-prazosin uptake in Caco-2 cells. Net fluorescence normalized to cell protein was measured following a 30-min incubation with 5 µM BODIPY-prazosin in the presence of varying concentrations of the indicated compounds from 0 µM (control, arbitrarily represented as 0.1 µM on the log-scale) to 100 µM. Results are expressed as the mean + SEM and are based on two separate experiments with *n* = 4 for each concentration per experiment. * indicates significantly different from the respective compound control (*p* < 0.05) from a one-way ANOVA followed by Tukey’s *t*-test; ** indicates significantly different from the control, 1, 3 and 10 µM conditions. Table insert summarizes the model-derived estimates of EC_50_ and E_max_ with associated coefficients of variation (% coefficient of variation) of the estimates.

BODIPY-prazosin uptake studies were conducted to discern any concentration-dependent effects of glyceollin and genistein on the uptake of this fluorescent BCRP substrate following a 30-min incubation with 5 µM of the substrate. Results of these uptake evaluations are summarized in Figure 6. Both Ko143 and glyceollin exposure resulted in concentration-dependent enhanced uptake of BODIPY-prazosin. As summarized in the accompanying table, the potency of glyceollin to produce this inhibitory effect on BODIPY-prazosin efflux by BCRP was similar to that of Ko143. As previously mentioned, the insolubility of glyceollin prevented the evaluation of concentrations >100 µM; thus, the E_max_ estimate for this compound is tentative given that it is well above the highest concentration evaluated.

In contrast to the glyceollin inhibitory effect, genistein exposure caused a significant concentration-dependent reduction in the uptake of the substrate, suggesting stimulation of BCRP-mediated efflux. The evaluation of the maximal genistein effect and EC_50_ is also summarized in the table of Figure 6. Also shown in Figure 6 is the lack of an effect of verapamil, a Pgp inhibitor, which supports that this assay is specific for BCRP. The genistein effect was limited (E_max_ of 68%), but reproducible over three experiments. Genistein’s effect on BCRP is similar to that observed with Pgp in our previous studies [24] and that reported by others [43]. In contrast to these effects in Caco-2, genistein has been reported to inhibit BCRP function in BCRP-overexpressing cell lines [44]. The different outcome is taken to be a consequence of the differential sensitivity of efflux transporter function to modulator concentration, substrate selection and the expression system [45].

## 4. Conclusions

Glyceollin demonstrates strong evidence that it inhibits both MRP2 and BCRP in Caco-2. Genistein exposure slightly increased BCRP activity, similar to its effects on Pgp in this intestinal cell line and may have also slightly enhanced MRP2 function. Since efflux transporters are important in determining drug absorption across the intestine and other important biological barriers (for example, the blood-brain barrier) and represent an important mechanism of cancer drug resistance in cancer cells, the findings with glyceollin are interesting and worthy of continued exploration for its ability to enhance drug delivery to cells.
